# Small Cell Cancer of the Bladder and Prostate: A Retrospective Review from a Tertiary Cancer Center

**DOI:** 10.7759/cureus.296

**Published:** 2015-08-07

**Authors:** Shahida Ahmed, Sam Neufeld, Tadeusz J Kroczak, Bashir Bashir, Naseer Ahmed, Piotr Czaykowski, Ibrahim Aljada, Rashmi Koul, Katie Galloway, Darrel E Drachenberg

**Affiliations:** 1 Radiation Oncology, CancerCare Manitoba, CancerCare Manitoba, University of Manitoba; 2 Faculty of Health Sciences, College of Medicine, University of Manitoba, Canada; 3 Urology, University of Manitoba, Canada; 4 Radiation Oncology, CancerCare Manitoba, University of Manitoba; 5 Medical Oncology, CancerCare Manitoba, University of Manitoba; 6 Pathology, University of Manitoba, Canada; 7 Epidemiology and Cancer Registry, CancerCare Manitoba; 8 Urology, St. Boniface Hospital, University of Manitoba, Canada

**Keywords:** small cell, genitourinary malignancies, chemotherapy, radiation, surgery, extrapulmonary small cell cancer

## Abstract

Background: Genitourinary small cell cancer (GUSCC) is a rare malignancy. Most of the published data on how to manage this malignancy is based on institutional experience. We undertook the current retrospective review to determine the outcome of the patients with GUSCC treated at CancerCare Manitoba, Canada over a period of 18 years.

Methods: The Manitoba Cancer Registry was used to identify patients with a confirmed pathological diagnosis of small cell cancer (SCC) of the bladder or prostate between January 1, 1995, and October 31, 2013.

Results: There were 42 patients identified, 28 bladder SCC (17 limited, 11 extensive stage) and 14 prostate SCC (one limited, 12 extensive, and one unknown stage). The median age was 70.7 years. There were 22 patients who were treated with chemotherapy and radiation, five received radiation only, four received chemo only, nine did not receive any treatment, one patient had surgery only, and one had surgery and radiation. The median and one-year overall survival for all patients was 10.7 months and 43%. The median and one-year overall survival of SCC of the bladder was 55.1 months and 71% for the limited stage and 10.1 months and 36% for the extensive stage. The median and one-year overall survival for extensive stage SCC of the prostate was 4.1 months and 17%. There was only one patient with limited stage SCC of the prostate who did not receive any treatment and died of progressive disease 11 months from diagnosis.

Conclusions: Our findings suggest that patients with limited stage SCC of the bladder can have a surprisingly good outcome with multimodality treatment. The outcome of the patients with extensive stage SCC of the bladder and prostate remains dismal and optimal therapeutic options have yet to be determined.

## Introduction

Extrapulmonary small cell cancer (EPSCC) is a rare malignancy and was first introduced in the medical literature as a separate clinicopathological disease entity in 1930 [1-3]. Bladder and prostate are one of the most common sites of EPSCC [4-6]. The pathogenesis of genitourinary small cell carcinoma (GUSCC) is poorly understood. It has been proposed that small cell cancer (SCC) develops from the metaplasia of other high-grade tumors, such as transitional cell carcinoma. However, this fails to explain the existence of EPSCC in the absence of other malignancies. Differentiation from a common pluripotent stem cell has also been proposed, which could account for both the existence of EPSCC in the absence of other tumor types and for their tendency to be present together [7-10]. SCC of the prostate is often associated with previously treated adenocarcinoma and may represent an escape mechanism from androgen deprivation therapy [11-12].

Within the genitourinary tract, the bladder and prostate are the most frequent locations with SCC, accounting for approximately 1% of all bladder tumors and 0.2% of all prostate cancers. This represents approximately 500 and 250 cases per year in the USA [13-14]. Most of the published data about this rare malignancy is based on institutional experience, and there are no randomized trials to address the optimum management of this unique entity [2, 4, 6, 15-17]. Historically, treatment has been based on the assumption that these cancers behave similarly to small cell lung cancer (SCLC), which is more common and has well-established treatment algorithms. Although small cell cancers share the same behavioral traits, EPSCC is a distinct entity with a unique natural history [14, 18]. This rare histological subtype is exceedingly aggressive and has a poor prognosis [5-6]. However, a number of studies have reported that cure is possible in patients with limited disease using surgery, chemotherapy, radiation, and various combinations of these treatment modalities [19-20].

We undertook the current retrospective review to determine the outcome of the patients with GUSCC treated at CancerCare Manitoba, Canada.

## Materials and methods

CancerCare Manitoba serves a population of approximately 1.4 million. The Manitoba Cancer Registry (MCR) was used to identify patients with a confirmed pathological diagnosis of bladder or prostate SCC between January 1, 1995 and October 31, 2013 with no radiological and pathological evidence of primary SCC of the lung. Patients diagnosed up to and including 2001 were coded using the International Classification of Diseases 9^th^ Clinical Modification ICD-9-CM and patients diagnosed after 2001 were coded using the International Classification of Diseases 10^th^ Revision, Canada. Both the MCR and CancerCare Manitoba patient charts were reviewed to collect data on clinical characteristics of the patients and survival. Statistical analyses were conducted using SAS™, Version 9.2 and the survival curves were created in STATA, Version 11.

The MCR has been recording staging information from 2004 onwards. The Collaborative Stage AJCC 6^th^ edition was used to determine the stages from 2004 to 2009 while the AJCC 7^th^ edition was used for staging from 2010 to 2013. Prior to 2004, a chart review was conducted to determine the patient stage. The Veterans Administration Lung Cancer Study Group Staging Classification was used to classify patients as having a limited or extensive stage. Patients with T1-T3, N0-N1, M0 were classified as having limited disease, whereas patients with T4 tumors or a distant metastasis were categorized as having extensive disease [21]. Response to treatment was determined according to RECIST version 1.1, and survival was calculated using the Kaplan–Meier method.

The University of Manitoba Biomedical Research Ethics Board approved this study (H 2006:001​).

## Results

### Clinical characteristics of the patients

A total of 42 patients with GUSCC were identified with 14 involving the prostate and 28 involving the bladder. The median age at diagnosis was 70.7 years (28-92 years). There were 38 males and four females. There were 28 bladder SCC (17 limited, 11 extensive stage) and 14 prostate SCC (one limited, 12 extensive, and one unknown stage). At presentation, 24 patients had hematuria, nine had a urinary obstruction, five had lower urinary tract symptoms (frequency, dysuria), three had systemic symptoms, and one had a raised PSA (Table 1). There were 12 patients with regional lymph node involvement, nine with liver, seven with lung, seven with bone, one with brain, two with seminal vesicles, one with rectal wall, one with colon, and one with bladder (primary prostate SCC) involvement (Table 1).

**Table 1:** Clinical characteristics of the patients

*Characteristics *	*N (%)*
Sex	
Male	38 (90)
Female	4 (10)
Age at Diagnosis	
Median	70.71
Range	28 - 92
Primary Site	
Bladder	28 (67)
Prostate	14 (33)
Stage	
Extensive Bladder Prostate	23 (55) 11 12
Limited Bladder Prostate	18 (43) 17 1
Unknown	1 (2)
Presenting Symptoms	
Hematuria Bladder Prostate	24 (57) 23 1
Urinary Obstruction	9 (21)
Dysuria and or Frequency	4 (2)
Raised PSA	1 (2)
Systemic Symptoms	3 (7)
Site of Metastases	
Regional nodes	12 (29)
Liver	9 (21)
Bone	7 (17)
Lung	7 (17)
Seminal Vesicle	2 (5)
Colon	1 (2)
Brain	1 (2)
Bladder	1 (2)
Rectal Wall	1 (2)

### Treatment and outcome of the patients

There were 22 patients, who were treated with chemotherapy and radiation, five received radiation only, four received chemo only, nine did not receive any treatment, one patient had surgery only, and one had surgery and radiation (Table 2).

**Table 2:** Treatment and outcome of the patients

Treatment			
Chemo + RT	22 (52)		
Surgery + RT	1 (2)		
RT Only	5 (12)		
Chemo Only	4 (10)		
Surgery Only	1 (2)		
No Treatment	9 (21)		
Outcome		Median Survival (95% CI)	1-year Overall Survival
All patients		10.7 Months (6.2, 13.5)	43%
Outcome by stage			
Limited Stage- Bladder		55.1 Months (8.2, 121.1)	71%
Extensive Stage -Bladder		10.1 Months (1.0, 14.0)	36 %
Extensive Stage-Prostate		4.1 Months (2.1, 11.1)	17%

**Figure 1:**
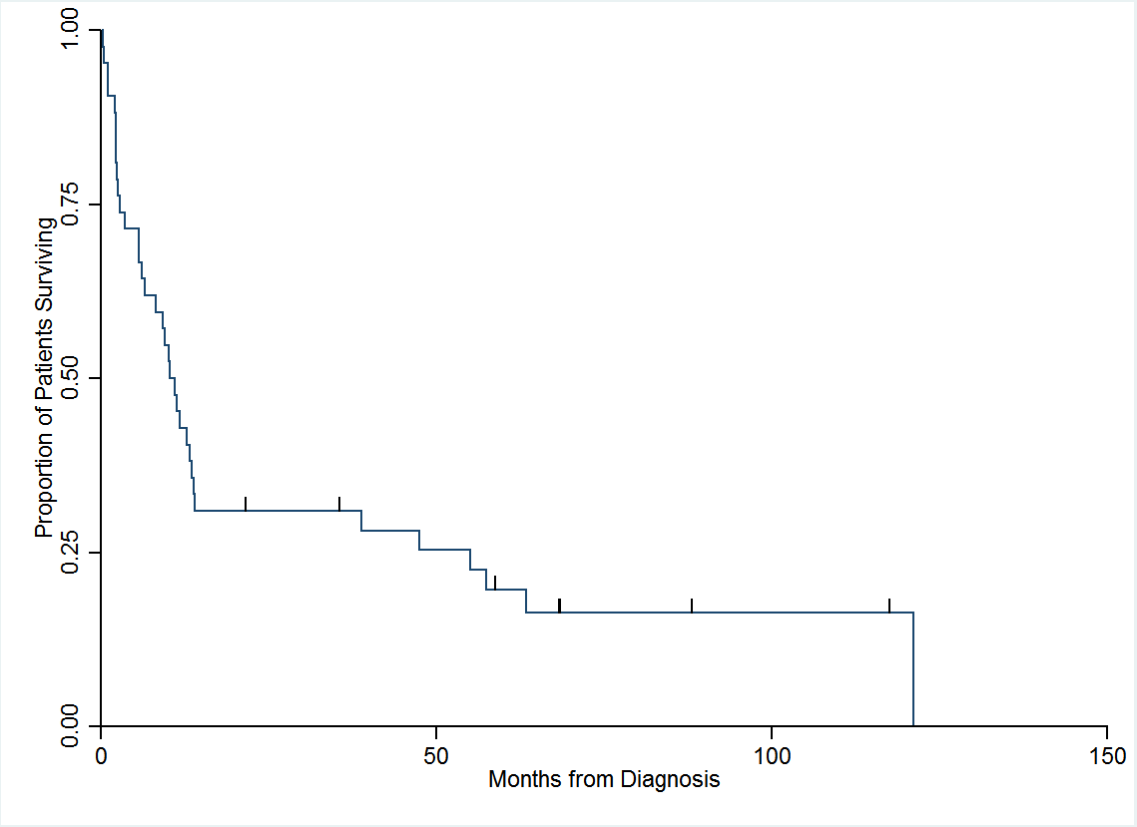
Overall survival of all the patients

**Figure 2:**
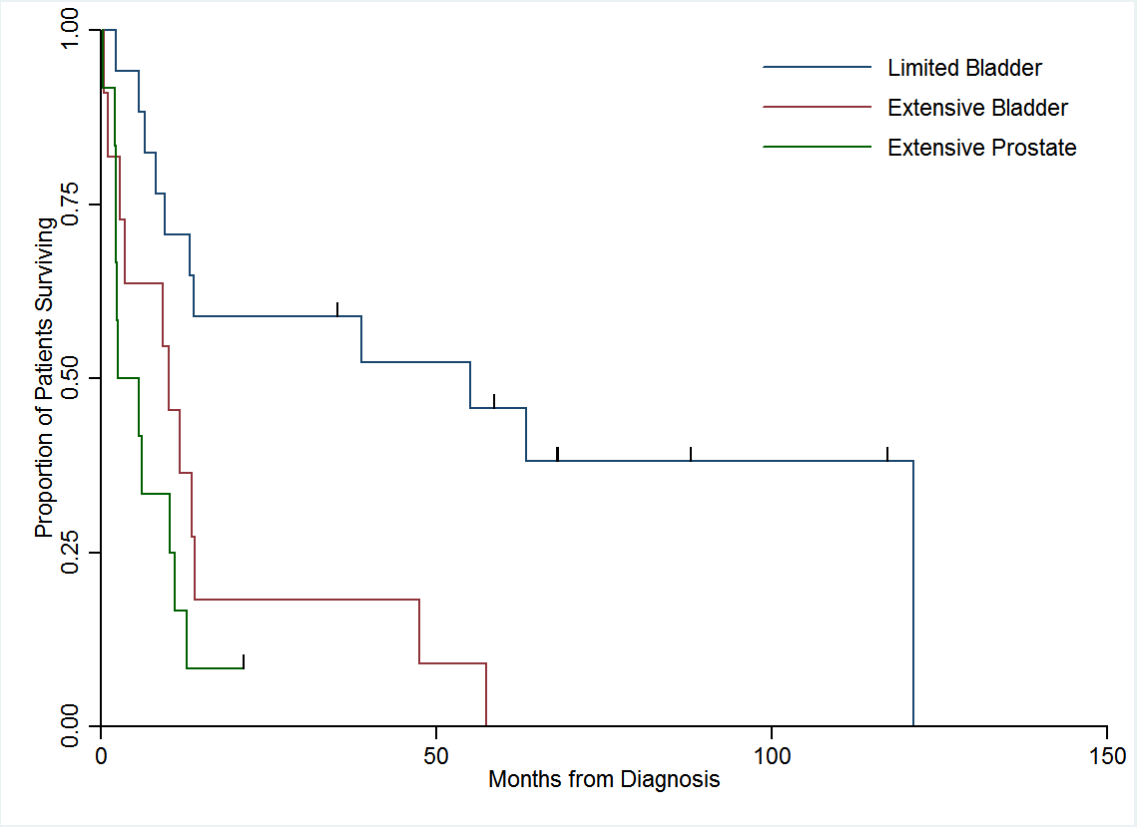
Overall survival of by stage and anatomical site

The median and one-year overall survival for all patients was 10.7 months (95% CI 6.2, 13.5) and 43% (Table 2, Figure 1). The median and one-year overall survival of SCC of the bladder was 55.1 months (95% CI 8.2, 121.1) and 71% for limited stage and 10.1 months (95% CI 1.0, 14.0) and 36% for extensive stage (p= 0.0067). There were six patients alive at the time of this review. The median and one-year overall survival for extensive stage SCC of the prostate was 4.1 months (95% CI 2.1, 11.1) and 17% (Table 2, Figure 2). There was only one patient with limited stage SCC of the prostate; hence, further analysis was not possible. This was an 89-year-old male who died of progressive disease 11 months from diagnosis without any treatment.

## Discussion

It remains a challenge for oncologists to decide the best management for their patients with EPSCC. In the current paper, we are presenting our institutional experience for the patients treated over a period of 18 years.

In our study, there were more patients with bladder SCC than patients with prostate SCC and more patients with extensive stage disease than with limited stage (23/42 vs. 18/42). This observation is in concordance to the previously published series [22-24]. Using the SEER database, Koay, et al. described a bleak outlook for those diagnosed with GUSCC. Among the 642 patients identified, the median survival was only 11 months, which is similar to the outcome in our study for the entire cohort.

Current treatment regimes are largely based on case reports and small retrospective studies with only a small number of Phase II trials available.

In a retrospective study of 25 patients, Quek, et al. found that patients receiving systemic chemotherapy as part of multimodal therapy had improved survivals compared to those treated with cystectomy alone [25]. They also noted that the difference in prognosis for those with and without lymph node involvement failed to reach statistical significance. This supported the findings of Koay, et al., which found that patients with local lymph node involvement typically followed a similar course to those without lymph involvement when compared to those with distant metastasis [14]. Bryant, et al. evaluated 11 patients treated with systemic chemotherapy followed by radiation to the bladder. The study did not group patients based on the extent of disease. Shorter than expected survival was found when compared to other studies with survival at three years of only 24%. Despite this poor overall survival, localized response was excellent with 73% of patients achieving a complete local response. The authors concluded that chemoradiation combined with transurethral resection of the bladder tumor (TURBT) remains a viable treatment option for bladder SCC and allows for preservation of the bladder and reduced morbidity [26]. The strong local response juxtaposed with the poor overall survival highlights the critical importance of systemic control. Meijer, et al. retrospectively reviewed a group of 27 patients with limited stage (Tx-4N0-1M0) bladder SCC treated with sequential chemotherapy and radiation following TURBT. Similar to the findings of Bryant, et al., patients who had an incomplete response to chemotherapy had a median cancer-specific survival of just 22 months, while the median cancer-specific survival in those with complete response was 52 months.  They concluded that response to chemotherapy was significantly correlated with survival and that bladder sparing sequential chemoradiation appears to be an acceptable option in the treatment of bladder SCC [27].

In one of the largest genitourinary SCC case series, Siefker-Radtke, et al. reviewed 46 patients who underwent radical cystectomy with curative intent; 21 of these received preoperative chemotherapy. This report included patients with Stage 3 and 4 disease without evidence of metastasis. The group treated with initial radical cystectomy had a median survival of 23 months with a five-year survival rate of 36%. Those who underwent preoperative chemotherapy achieved a five-year survival rate of 78% with surgical down-staging in 12 of 21 patients treated with cisplatin. They concluded that a combination of systemic chemotherapy and localized management is required to achieve optimal results. They further argued that due to the risk of recurrent urothelial cell carcinoma, radical cystectomy is the preferred method of local control [28]. Siefker-Radtke, et al. followed up their 2004 study with a Phase II clinical trial. They reported surgical down-staging (≤ pT1N0M0) in 14 of 18 patients treated with four cycles of neoadjuvant chemotherapy, alternating between combinations of cisplatin and etoposide, and ifosfamide and doxorubicin. In keeping with their earlier results, the non-metastatic arm of this trial achieved an overall survival of 58 months. Within the metastatic arm, they reported a near complete response to this chemotherapeutic regimen in 8 of 12 patients. In spite of this apparent response, the relapse rate was high and the overall survival was only 13.3 months. The authors concluded that this disease is both difficult to control and to characterize based on clinical stage [29]. This study confirmed their 2004 conclusion that multimodal therapy consisting of systemic chemotherapy followed by a localized therapy is necessary for optimal results.

Another more recent study by Lynch, et al. further delineated the survival benefit associated with neoadjuvant chemotherapy. In their study of 95 patients treated surgically, they observed a stark contrast in the outcome between those treated with neoadjuvant chemotherapy with a median survival of 159.5 months and those treated with initial cystectomy with a median survival of 18.3 months. Patients who were poor surgical candidates or who refused cystectomy typically received radiation or chemo-radiation; no significant survival benefit was observed between this group and those receiving cystectomy. Authors of this study concluded that surgical management provides optimal control and chemoradiation is a useful alternative in patients unwilling or unable to undergo surgery [20] This is by far the longest reported median survival which adds further evidence that combination therapy is most often needed to achieve a durable response. Follow-up time used in this study was relatively long and extended from 1985 to 2012.

In 2013, Morretto, et al. published the first Canadian guidelines for the treatment of small cell bladder cancer. Their recommendations varied based on disease stage, suggesting that limited disease be treated with chemotherapy in addition to either radiation therapy or radical surgery while advanced disease be treated with chemotherapy alone [30].

Prostate SCC is more likely to present with advanced disease when compared to bladder SCC. Not surprisingly, it also has a worse prognosis [31]. Tagawa, et al. recently described a link between previously treated adenocarcinoma of the prostate and the development of neuroendocrine disease not responsive to androgen deprivation therapy. Due to the advanced stage at presentation and low overall incidence, treatment data for prostate SCC is even more limited than bladder SCC.  Similarly to bladder SCC, chemotherapy is an important part of multimodal treatment regimes required to achieve favorable treatment outcomes for prostate SCC [24]. Unfortunately, due to the fact that PSA values do not correlate with SCC, early detection is extremely difficult.

As evident in the published literature, EPSCC carries a high risk of systemic relapse and multimodal therapy with cisplatin and etoposide chemotherapy appears to be the most widely accepted treatment modality [24, 31-32]. Most studies recommend combination therapy centered on systemic platinum-based chemotherapy and local therapy with either radiation therapy or radical cystectomy [33-34]. The majority of patients in our study presented with extensive stage disease and, independent of their stage, were treated with chemotherapy (26/42) with or without local therapy. Further, in our patients, radiation was employed more frequently and only one patient underwent surgery.

In a large randomized trial of SCLC evaluating sequencing of cisplatin and etoposide, median survival of 20 months for the limited and 11.1 months for the extensive stage was reported [35]. Similarly in our study, limited stage SCC of the bladder had a better outcome than patients with extensive stage SCC of the bladder and prostate. Similar response to cisplatin-based chemotherapy may indicate a common biology of SCLC and EPSCC [14, 18].

In the current study, only 2% of the patients presented with brain metastasis. This is consistent with the published data indicating a lower rate of brain relapse in EPSCC as compared to SCC of the lung [2, 15-16, 24]. Prophylactic cranial radiation (PCI) is routinely used for limited stage SCC of the lung. Considering the very low rate of relapse in the brain in patients with EPSCC, PCI may not be indicated routinely [24].  

### Limitations of the study

This is a retrospective review with a small sample size, including only one patient with limited stage SCC of the prostate. Patients have been treated over almost two decades possibly without using a consistent staging system. 

 

## Conclusions

Genitourinary SCCs are aggressive malignancies with a high propensity to metastasize. Our findings suggest that limited stage bladder SCC patients can have a surprisingly good outcome with multimodality treatment centered around platinum-based chemotherapy. The outcome for prostate SCC and extensive stage bladder SCC remains dismal, and optimal therapeutic options have yet to be determined. Future advances in understanding the molecular oncologic pathways at work in the pathogenesis of these neuroendocrine tumors may hold promise for targeted therapeutic approaches to improve outcomes. Authors suggest a multicenter prospective study to standardize the treatment and improve the outcome of this rare and lethal disease.
